# Inducible gene expression system by 3-hydroxypropionic acid

**DOI:** 10.1186/s13068-015-0353-5

**Published:** 2015-10-20

**Authors:** Shengfang Zhou, Satish Kumar Ainala, Eunhee Seol, Trinh Thi Nguyen, Sunghoon Park

**Affiliations:** School of Chemical and Biomolecular Engineering, Pusan National University, San 30 Jangeon-dong, Geumjeong-gu, Busan, 609-735 Republic of Korea; Department of Biochemical Engineering, College of Pharmaceutical and Life Sciences, Changzhou University, Changzhou, 213164 China

**Keywords:** 3-Hydroxypropionic acid, LysR, Inducible, *hpdH*, *mmsA*, *hbdH*

## Abstract

**Background:**

3-Hydroxypropionic acid (3-HP) is an important platform chemical that boasts a variety of industrial applications. Gene expression systems inducible by 3-HP, if available, are of great utility for optimization of the pathways of 3-HP production and excretion.

**Results:**

Here we report the presence of unique inducible gene expression systems in *Pseudomonas denitrificans* and other microorganisms. In *P. denitrificans*, transcription of three genes (*hpdH*, *mmsA* and *hbdH*-4) involved in 3-HP degradation was upregulated by 3-HP by the action of a transcriptional regulator protein, LysR, and a *cis*-acting regulatory site for LysR binding. Similar inducible systems having an LysR transcriptional regulator were identified in other microorganisms that also could degrade 3-HP. A docking study showed that the 3-HP binding pocket is located between the N-terminal helix-turn-helix motif and the C-terminal cofactor-binding domain.

**Conclusions:**

This LysR-regulated 3-HP-inducible system should prove useful for control of the level of gene expression in response to 3-HP.

**Electronic supplementary material:**

The online version of this article (doi:10.1186/s13068-015-0353-5) contains supplementary material, which is available to authorized users.

## Background

3-Hydroxypropionic acid (3-HP) is an important platform chemical. It can be converted to acrylic acid, acrylamide, 1,3-propanediol, malonic acid, and others. It can also be used for synthesis of biodegradable polymer [1–3]. Biological production of 3-HP from glycerol has been successfully demonstrated in several bacteria such as *Escherichia coli*, *Klebsiella pneumoniae* and *Pseudomonas denitrificans*, which are engineered to (over)express glycerol dehydratase (GDHt, coenzyme B_12_ -dependent), glycerol dehydratase reactivation factors (GdrAB), and/or aldehyde dehydrogenase (ALDH, NAD^+^-dependent) [4–8]. Some recombinant strains such as *E. coli* W DUBGK have produced >40 g/L of 3-HP in 48 h [9], but further increases of 3-HP production have been challenging. The 3-HP pathway enzymes GDHt and ALDH have been shown to be unstable and to lose their activities during prolonged fermentation, especially in the late stage [9].

One important mechanism of GDHt activity loss is so-called suicidal inactivation. Coenzyme B_12_, the cofactor of GDHt, is irreversibly damaged during dehydration reaction of the substrate glycerol to 3-hydroxypropionaldehyde (3-HPA) [10]. This inactivation is aggravated in the presence of oxygen. To alleviate this mechanism-based inactivation, Yamanishi et al. [11] developed mutant GDHt by site-directed mutagenesis. Some of the mutant enzymes exhibited improved stability, but the activity was significantly lower than that of the wild type. Toxic intermediate 3-HPA also causes enzyme instability. It was observed that, when GDHt or ALDH was incubated with 3-HPA, its activity declined in a dose-dependent manner (unpublished data). Aldehyde is known to react with amino acid residues lysine, cysteine and histidine by targeting the ε-amino group (NH_3_^+^), the sulfhydryl group (-SH) and the imidazole group, respectively [12–15]. Serious efforts to stabilize enzymes against aldehydes by site-directed or random mutagenesis have been made, but with only limited success [13, 16].

One alternative approach to solve the instability problem in 3-HP production is to synthesize new and active enzymes continuously during the entire period of cultivation. This is difficult, especially in the late period of fermentation, where cell growth is very slow and target products often accumulate at levels toxic enough to interfere with cellular metabolisms. However, if there exists a dynamic 3-HP-inducible promoter, gene expression for the aforementioned, unstable GDHt and ALDH enzymes can be stimulated and their enzymatic activities can be maintained at high levels, even in the late period of 3-HP production. To this end, we attempted to screen such promoters in 3-HP-degrading *P. denitrificans*, and identified two of them in this specific strain. After analyzing the 3-HP-inducible system’s gene arrangement, including transcriptional activator proteins, promoter sequences and structural genes under the control of such 3-HP-inducible promoters, similar 3-HP-inducible gene expression systems were searched and identified in other microorganisms. The existence of 3-HP-inducible gene expression systems in many of these microbes was confirmed by measurement of the transcriptions of the genes under the control of the 3-HP-inducible promoters.

## Results and discussion

### Screening of 3-HP-inducible promoters in *P. denitrificans*

**Fig. 1:**
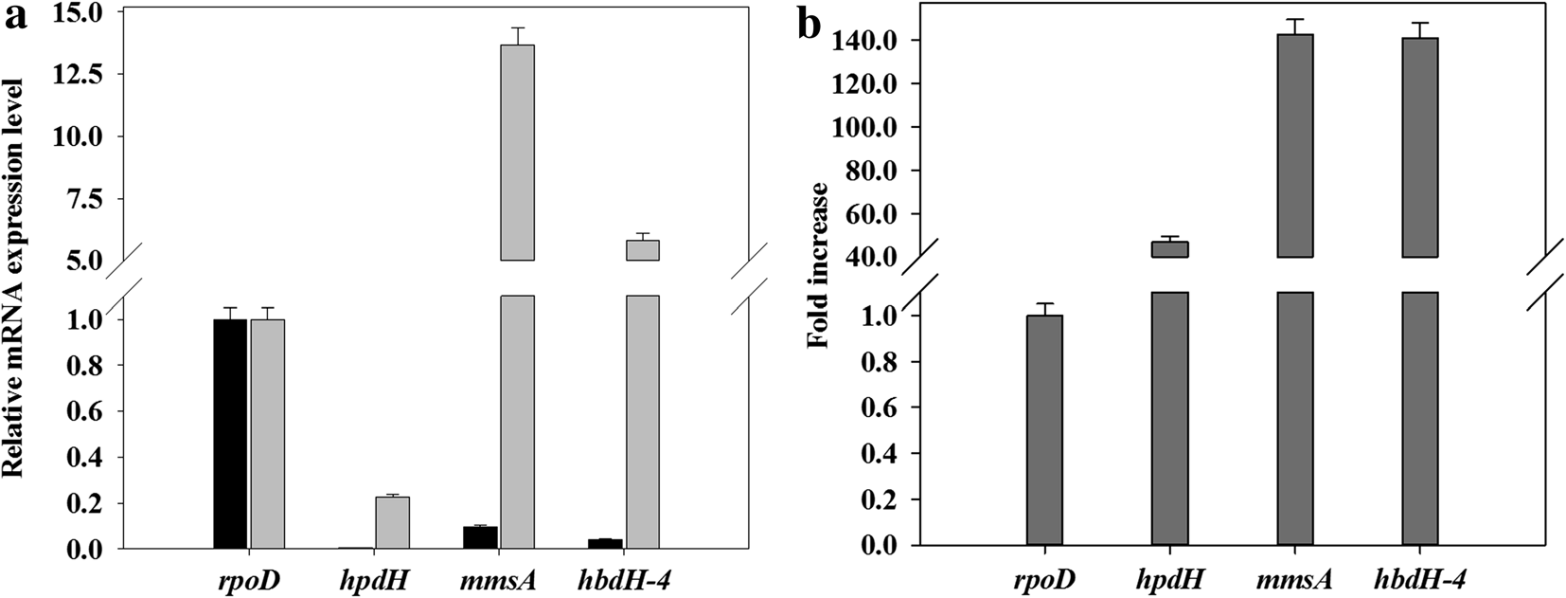
**a** Relative mRNA levels and **b** fold increase of 3-hydroxypropionate catabolism genes in wild-type *P. denitrificans* ATCC13867. *P. denitrificans* cells were cultivated in M9 minimal medium containing 5 g/L sodium gluconate. At OD_600_ of ~0.4–0.5, 3-HP was supplemented at 25 mmol/L (*gray bar*) or 0 mM (*black bar*), and cells were harvested after a further 2 h cultivation. The fold increase is indicated by the dark gray bar. The standard deviation of the mRNA-level measurements was <10 %. The mRNA levels were compared with those of the reference gene, *rpoD*

3-Hydroxypropionic acid (3-HP) is a carbon compound not commonly encountered in the natural environment; neither its use as a carbon substrate nor its biological degradation has been adequately elucidated. Recently, we found that *P. denitrificans* can grow on 3-HP as carbon source and, further, that it degrades 3-HP under non-growing conditions in the presence of oxygen [7, 8]. Genome sequence and metabolite analysis by gas chromatography/mass spectrometry [7] suggested that 3-HP is metabolized in *P. denitrificans* sequentially via two major enzymes, a putative 3-hydroxypropionate dehydrogenase (HpdH) and (methyl)malonate-semialdehyde dehydrogenase (MmsA) [8, 17, 18], and converted to acetyl-CoA. In activity assay with purified enzymes, a putative 3-hydroxyisobutyrate dehydrogenase (HbdH-4) also exhibited 3-HP degradation [19]. Because the degradation activity of 3-HP in *P. denitrificans* was found to increase greatly when cells were exposed to 3-HP [8], we hypothesized that the expressions of these enzymes are induced by 3-HP. Thus, transcription of three chosen 3-HP catabolic genes, *hpdH*, *hbdH*-4 and *mmsA*, was examined by quantitative RT-PCR with and without exposure to 3-HP (Fig. 1). The housekeeping gene *rpoD*, encoding sigma factor 70, was used as a reference. As expected, expression of all three putative 3-HP catabolic genes was enhanced markedly upon exposure to 3-HP: 47-fold for *hpdH*, 141-fold for *hbdH*-4, and 142-fold for *mmsA*. This indicates that transcription of the three genes is induced by 3-HP. The levels of transcription for *mmsA* and *hbdH*-4 were much higher than that for *hpdH*, suggesting that the promoter strength of the first two genes is much stronger than the other.

### Analysis of 3-HP-inducible gene expression system

**Fig. 2:**
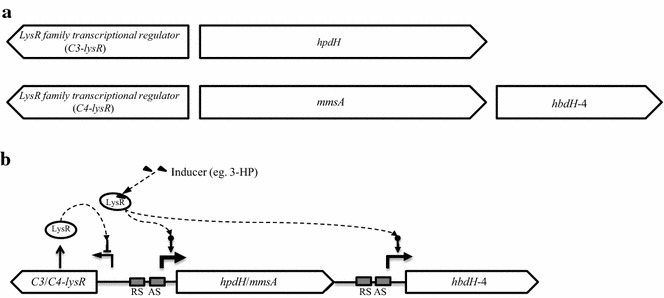
Genetic arrangement of 3-HP catabolism genes in *P. denitrificans* and analysis of LysR regulation system. **a** 3-HP catabolic genes (*hpdH*, *hbdH*-4 and *mmsA*) divergently located relative to l*ysR*-family transcription regulatory gene. **b** Hypothetical scheme of typical structure of divergent promoter regulated by LysR-family transcriptional regulator. *RS* regulatory site, *AS* activation site, *Conserved motif* T-N_11_-A or TTA-N_7/8_-GAA

**Fig. 3:**
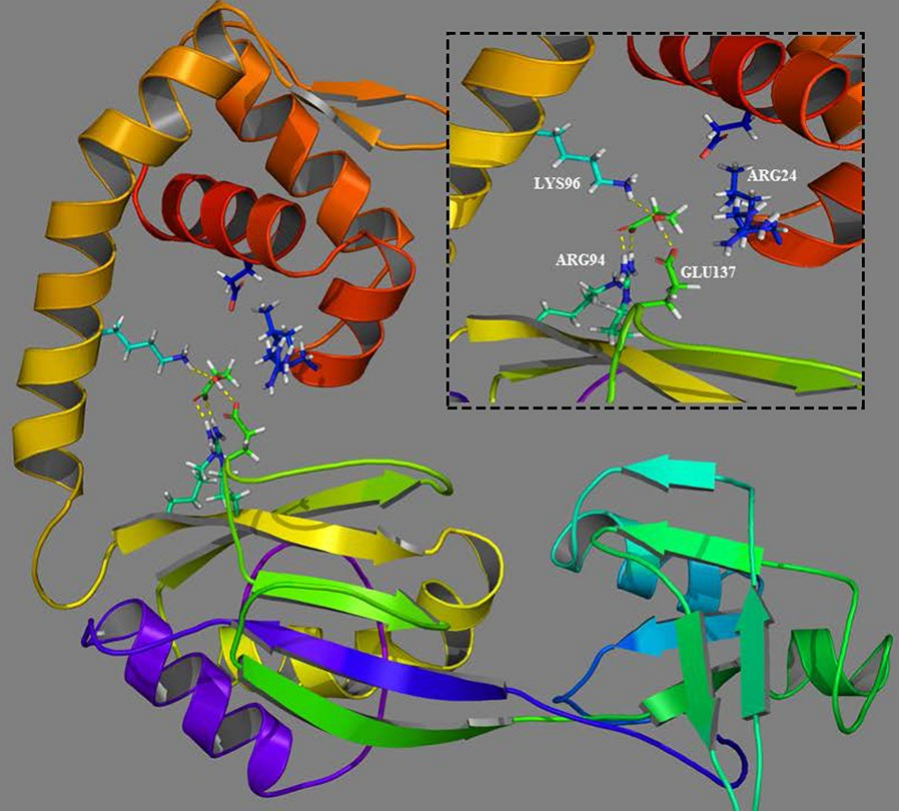
Predicted three-dimensional (3D) structure and docking analysis of C4-LysR in *Pseudomonas denitrificans* ATCC 13867. The *inset* shows the active-site pocket amino acid interactions with 3-HP. The hydrogen bonds are represented by the *yellow dotted lines*

To unravel the details of the 3-HP-induced gene expression systems, the genetic arrangement of the operons for *hpdH*, *hbdH*-4 and *mmsA* in *P. denitrificans* was analyzed (Fig. 2). The three genes were found to exist in two separate operons, *hpdH* in one operon (the C3 operon, hereafter) and *hbdH*-4 and *mmsA* in another (the C4 operon). Located just before each of the C3 and C4 operons, there was an oppositely oriented gene encoding a LysR-type transcriptional regulator (LTTR) (Fig. 2a), which suggests the possible involvement of LysR protein in the regulation of the expressions of those genes. Additionally, this suggests that the transcription of LysR regulator protein is also self-regulated, though negatively. LysR protein is known to be composed of a DNA-binding domain (helix-turn-helix motif) at the N-terminus and a cofactor-binding domain at the C-terminus [20]. When binding to DNA, LysR protein forms a homodimer and targets two specific binding sites, a regulatory binding site, which has the conserved T-N_11_-A/TTA-N_7/8_-GAA motif, and an activation binding site (near the -35 RNA polymerase binding site) (Fig. 2b). The tetramer of LysR, formed through protein–protein interaction between two LysR homodimers, is known to lead to conformational changes in DNA and, thereby, to enhancement of RNA polymerase binding in the promoter sequence. The conserved T-N_11_-A/TTA-N_7/8_-GAA motif also was identified in both operons. The structure of the putative LysR protein for the C4 operon was analyzed as well (see Fig. 3 for more details). Features similar to those found in other LysR proteins, namely a DNA-binding helix-turn-helix motif, a substrate-binding motif and a linker connecting the two, were identified in C4-LysR. The structural characteristics of the putative LysR, the existence of the LysR-binding T-N_11_-A/TTA-N_7/8_-GAA motif DNA sequence in the intergenic region, as well as the RT-PCR results shown in Fig. 1, strongly support the conclusion that gene transcription in the C3 and C4 operons is induced by the LysR-3-HP complex.

### Virtual screening of 3-HP-inducible gene expression systems

**Fig. 4:**
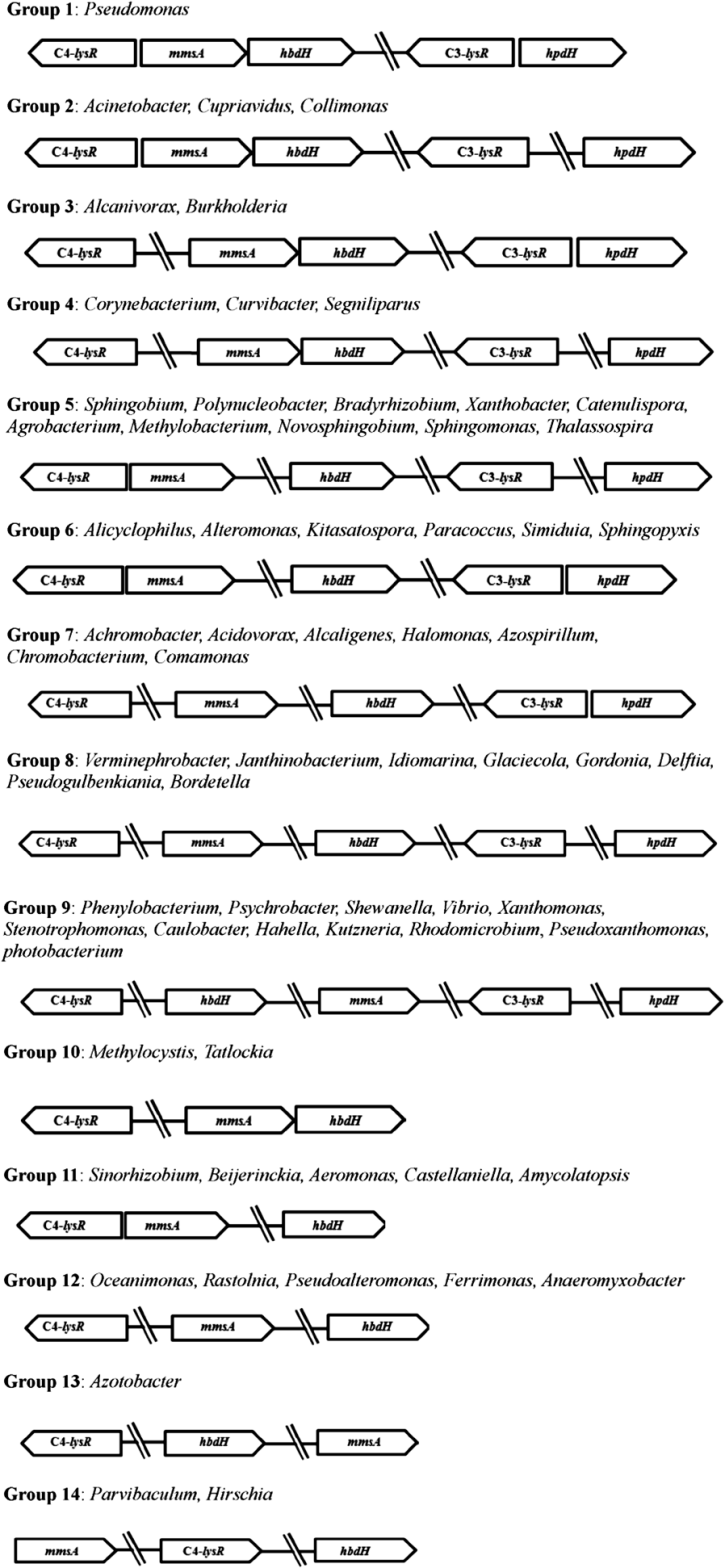
Gene organization of C3 and C4 operons involved in 3-HP degradation pathway in various microbial genera

The LTTR-mediated 3-HP-inducible gene expression systems were screened in other microorganisms using homology searches of various public and private databases such as NCBI (nr, refseq_protein, and env_nr), MBGD (Microbial Genome Database), and SEED. The most conserved components of the C3 and C4 operons from *P.denitrificans*, HpdH (C3-LysR) and HbdH-4 (C4-LysR), were used as primary queries. The 3-HP-inducible systems existed in more than 53 genera among the members of the sequenced bacterial species; some of them had both C3 and C4 operons, while others C4 operon only. The 53 genera were arbitrarily grouped into 14 based on (1) their gene organization and (2) the existence of the C3 operon (Fig. 4). A comparison among the various organisms revealed that the genetic organizations and compositions of the LysR systems differed significantly. Notably for example, the C4 system was more commonly present than the C3. Interestingly too, in many microorganisms, the genes encoding a DNA-binding regulator protein (C3- or C4-LysR) were located just before the corresponding C3 or C4 operon and oppositely oriented, indicating that transcription of the LysR regulator protein is self-repressed, as in the case of *P. denitrificans* (see Fig. 2). However, in other microorganisms, the gene encoding LysR was located remotely from the C3 or C4 operon and in the same orientation. Further studies are required to understand the significance of the different LysR gene arrangements among various genera.

**Fig. 5:**
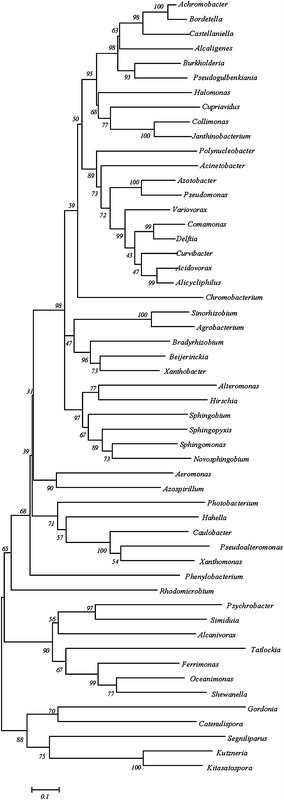
Evolutionary relationships of C4-LysR in various microbial genera. The location of *Pseudomonas* in the tree is marked. The* numbers* on the tree branches represent the measure of statistical confidence in each branch of the tree; genera with confidence levels below 30, generally indicative of lower confidence, were eliminated

A phylogenetic tree was generated to analyze the proximities among the C4-LysR variants (Fig. 5). The C4-LysR of *Pseudomonas* was close to that of *Azotobacter* but far from those of *Kitasatospora, Kutzneria, Segniliparus, Catenulispora* and *Gordonia*. The multiple sequence alignment for the helix-turn-helix regions of C3- and C4-LysR exhibited a high amino acid sequence homology (data not shown). Moreover, the amino acid sequences of the enzymes HpdH, HbdH and MmsA appeared to be significantly conserved across all of the species studied (Additional files 1 and 2: Tables S1 and S2). This indicates that the 3-HP-inducible LysR system and the operons regulated by LysR are widely distributed across a broad range of microorganisms.

### Molecular modeling and docking

Wek et al. reported that LysR-type protein binds to operator sites and directs a conformational change in the structure of the promoter–operator DNA complex [21]. When bound by inducer molecules, LysR protein enhances the recruitment of RNA polymerase to the promoter without affecting the occupancy of the LysR protein at the operator site. However, the ligand-associated conformational changes in LysR protein and their effect on the activity of LysR have not been fully elucidated. For detailed understanding of the structural and functional characteristics, protein modeling was performed for C4-LysR and its interaction with 3-HP (protein–ligand interaction). Because the crystal structure of C4-LysR is not available, multiple template threading was employed (see “Methods”). The predicted model was evaluated using the RAMPAGE tool, by calculating the main-chain RMSD (Root Mean Square Deviation) with reference to its template structure (3SZP) and their amino acid distribution.

Among the various docking poses generated, the best one showed the lowest Glide docking score (empirical scoring function) of −5.01 kcal/mol with three hydrogen bonds and a hydrophobic contact, which indicated the high reliability of the simulation [22]. Several intermolecular interactions were identified between C4-LysR and the 3-HP molecule: three amino acid residues (ARG94, LYS96 and GLU137) present in the substrate-binding domain showed hydrogen bonding with 3-HP, while ARG24 located in the helix-turn-helix domain, exhibited a hydrophobic interaction (Fig. 3). The steric hindrance caused by the binding of 3-HP to the active site of C4-LysR might be responsible for protein dimerization and DNA relaxation. Thus, increasing the RNA polymerase (RNAP) binding at the promoter site would effectively activate transcription of the target genes.

### Resting-cell degradation of 3-HP and 3-HP-dependent transcription activation of C3 and C4 operons in selected microorganisms

As in the case of *P. denitrificans*, the genes in the C3 and C4 operons in the microorganisms presented in Fig. 4 were expected to get involved in 3-HP degradation and/or assimilation. To confirm this, 3-HP degradation under non-growing conditions was examined for 10 selected microorganisms (Table 1). Cells were cultured in nutrient media containing 25 mM 3-HP to mid-log period, harvested by centrifugation, and resuspended in 100 mM potassium phosphate buffer (pH 7.0) containing 25 mM 3-HP. After 24 h incubation at 30 °C, the amount of degraded 3-HP was determined. All 10 of the microorganisms degraded 3-HP, though the amounts varied. Four strains, *Pseudomonas protegens* CHA0, *Pseudomonas fluorescence* A506, *Alicycliphilus denitrificans* and *A. avenae* subsp. *citrulli*, showed high degradation yields similar to that of *P. denitrificans*. In contrast, *Pseudomonas knackmussii* B13, *Pseudogulbenkiania* sp. and *Variovorax paradoxus* exhibited up to 50 % lower degradation yields compared with *P. denitrificans*.

**Table 1:** Bacterial strains used in this study and their 3-HP degradation

Strain	Source	3-HP degraded (mM)^a^
*Pseudomonas denitrificans* ATCC13867	KCCM, Korea	20.5
*Pseudomonas knackmussii* B13	DSM, Germany	7.4
*Pseudomonas protegens* CHA0	DSM, Germany	25.2
*Pseudomonas fluorescens* A506	ATCC, America	24.4
*Alicycliphilus denitrificans*	DSM, Germany	20.6
*Pseudogulbenkiania* sp.	DSM, Germany	7.4
*Collimonas arenae*	DSM, Germany	16.1
*Acidovorax* sp.	DSM, Germany	16.6
*Achromobacter xylosoxidans*	KCCM, Korea	18.4
*Acidovorax avenae* subsp. *citrulli*	KCTC, Korea	20.4
*Variovorax paradoxus*	KCTC, Korea	9.9

^a^The 3-HP degradation was measured under non-growing conditions and the amount was calculated between 0 and 24 h

The transcription levels of the 3-HP catabolic genes (*hpdH*, *hbdH*, *mmsA*) in the 10 microorganisms also were determined, after growing them in the absence or presence of 25 mM 3-HP (Table 2). Six showed improved transcription for the three 3-HP catabolic genes, with the exception of *Acidovorax* sp. for *hpdH*. As in the case of *P. denitrificans*, transcription of *hpdH* was lower than that of *mmsA* or *hbdH,* indicating that the promoter strength of the former is weaker than the others. In three of the microorganisms (*Collimonas arenae*, *Achromobacter xylosoxidans*, *V. paradoxus*), the mRNA of the 3-HP catabolic genes was not amplified at all, which suggested that the degenerate primer sequences, designed based on NCBI database, did not match the original gene sequence (Additional file 3: Table S3). With *P. protegens* CHA0, total RNA could not be isolated using standard RNA isolation procedures (see “Methods”). It should be noted that the fold increases in transcription of three genes in the six microorganisms (*P. knackmussii* B13, *P. fluorescence* A506, *A. denitrificans,Pseudogulbenkiania* sp., *Acidovorax* sp. and *A. avenae* subsp. *citrulli*) are generally lower compared to those of *P. denitrificans* (Table 2). In *P. denitrificans*, the fold increase was in the range of 47–142, while that in the other six microorganisms was <37. The reason for this difference is not clear, though we suspect that culture conditions, including medium composition, could have affected expressions of C3- and C4-LysR and the catabolic genes under their control. Nevertheless, the enhanced transcription of the putative 3-HP catabolic genes, along with the resting-cell 3-HP degradation, strongly suggests that the 10 microorganisms tested (Table 1) and, probably, most of other microorganisms listed in Additional files 1 and 2: Tables S1 and S2 have 3-HP-inducible C3 and/or C4 operons similar to those of *P. denitrificans*. Further in vivo and in vitro studies to elucidate the detailed mechanism of 3-HP-inducible systems are in progress.

**Table 2:** Fold increases of mRNA expression levels of *hpdH*, *mmsA* and *hpdH* by induction of 3-HP in various microorganisms

Strain	Fold increase		
	*hpdH*	*mmsA*	*hbdH*
*Pseudomonas denitrificans* ATCC13867	47.0	142.4	140.9
*Pseudomonas knackmussii* B13	10.4	3.9	4.2
*Pseudomonas protegens* CHA0^a^	–	–	–
*Pseudomonas fluorescens* A506	24.6	9.1	17.8
*Alicycliphilus denitrificans*	6.3	37.3	20.2
*Pseudogulbenkiania* sp.	1.6	2.4	100.6
*Collimonas arenae*	ND	ND	ND
*Acidovorax* sp.	0.7	6.8	5.9
*Achromobacter xylosoxidans*	ND	ND	ND
*Acidovorax avenae* subsp. *citrulli*	7.7	33.1	14.0
*Variovorax paradoxus*	ND	ND	ND

*ND* not detectable

^a^Failed to isolate RNA

## Conclusions

Unique transcriptional activator proteins and promoters that respond to 3-HP were studied. In the presence of 3-HP, two different LysR-family transcriptional regulators, designated C3- and C4-LysR, respectively, were found to stimulate the transcription of the catabolic genes *hpdH, hbdH* and/or *mmsA* involved in 3-HP degradation. The inducible systems were common to many microorganisms: more than 53 genera, according to the genome sequence databases. The present molecular modeling and docking studies suggested that, in C4-LysR, the four amino acid residues ARG94, LYS96, GLU137 and ARG24 interact with 3-HP and activate the LysR regulator protein. The 3-HP-inducible systems promise to be of a great utility to the development of gene expression systems that are regulated by 3-HP.

## Methods

### Materials

*Pseudomonas denitrificans* ATCC 13867 and *Pseudomonas fluorescens* A506 were purchased from ATCC (America). *A. xylosoxidans* were obtained from KCCM (Korea). *A. avenae sub* sp. *citrulli* and *V. paradoxus* were obtained from KCTC (Korea). *P. knackmussii* B13, *P. protegens*, *A. denitrificans*, *Pseudogulbenkiania* sp., *C. arenae* and *Acidovorax* sp. were acquired from DSM (Germany). The primers were synthesized by Cosmo Genetech Co. Ltd (Seoul, Korea). 3-HP was purchased from Tokyo Kasei Kogyo Co. Ltd., Tokyo, Japan (TCI America, Portland, OR). Yeast extract (Cat. 212750), tryptone (Cat. 211705), trypticase soy broth (Cat. 211768), and peptone (Cat. 211921) were supplied by Difco (Becton–Dickinson; Franklin Lakes, NJ). Unless indicated otherwise, all of the other chemicals and enzymes were purchased from Sigma-Aldrich (St. Louis, MO).

### Resting-cell degradation of 3-HP

Non-growing cell experiments were performed to examine 3-HP degradation by some of the bacteria strains listed in Table 1. Active cells were prepared by growing the strains each in its own specified enriched nutrient medium using 250 mL Erlenmeyer flasks with 50 mL working volume under aerobic conditions with 200 rpm agitation speed in an orbital incubator shaker. The nutrient medium for the *P. fluorescence* strain contains the following components per liter: peptone, 20 g; glycerol, 10 mL; K_2_HPO_4_, 1.5 g; MgSO_4_·7H_2_O, 1.5 g; 3-HP, 25 mmol. For the *P. knackmussii*, *P. protegens*, *Pseudogulbenkiania* sp., *A. xylosoxidans*, *V. paradoxus, Acidovorax avenae* subsp. *citrulli*, *C. arenae* and *A. denitrificans* strains, the nutrient medium contains the following components per liter: peptone, 5 g; beef extract 3 g; 3-HP, 25 mmol. For the *Acidovarax* sp. strain, trypticase soy broth containing 25 mmol/L was used. The cultures were conducted at 30 °C, obtaining ~1–1.5 OD_600._ The cells were then harvested and centrifuged at 5000 rpm for 10 min. The resultant pellet was washed with 100 mM potassium phosphate buffer (pH 7.0) and resuspended in the same buffer supplemented with 25 ± 2 mmol/L 3-HP. The cells were then harvested and centrifuged as above noted, subjecting them to non-growing 3-HP degradation experimentation. After 24 h cultivation, the samples were withdrawn to determine the 3-HP concentrations.

### RNA extraction and Real-time PCR

The *P. denitrificans* ATCC 13867 strain was grown in M9 minimal medium containing 5 g/L sodium gluconate; the other microorganism strains listed in Table 1 were grown in a specified nutrient medium. The cells were cultivated under the aerobic condition at 37 or 30 °C and 200 rpm in an orbital incubator shaker. 3-HP at 0/25 mM was supplemented at OD_600_ of ~0.4–0.5. After a further 2 h cultivation, approximately 5 × 10^8^ cells were collected and centrifuged at 5000×*g* for 10 min. The cell pellets were immediately resuspended in 500 μL of RNAlater solution (Ambion, UK). RNA was extracted using a total RNA isolation kit (Macherey–Nagel, Germany). One microgram of total RNA was employed to synthesize the first-strand cDNA in a 20 µL reaction using the SuperScript III first-strand synthesis system (Invitrogen, USA). A real-time PCR analysis was performed, according to the SYBR green method, in a 20 µL reaction volume using the StepOne Real-Time PCR system (Applied Biosystems, USA). The PCR efficiencies of all of the primers were experimentally determined and found to be suitable for reliable copy-number quantification. The relative quantification for each of the mRNA levels was calculated using the ΔΔ*C*_t_ method as described previously [23]. All of the assays were performed in duplicate, and a template-less reaction template was used as a negative control.

### Phylogenetic tree

The evolutionary history was inferred using the Neighbor-Joining method [24], which proceeds as follows: a bootstrap consensus tree inferred from 1000 replicates is taken to represent the evolutionary history of the taxa analyzed [25]; branches corresponding to partitions reproduced in less than 50 % of the bootstrap replicates are collapsed; the percentages of replicate trees in the associated taxa clustered together in the bootstrap test (1000 replicates) are shown next to the branches; the tree is drawn to scale, with branch lengths (in the same units as those of the evolutionary distances) used to infer the phylogenetic tree. The evolutionary distances were computed, using the Poisson correction method [26], in units of the number of amino acid substitutions per site. Evolutionary analyses were conducted in MEGA5 [27].

### Protein modeling and docking of C4-LysR

A model three-dimensional (3D) C4-LysR structure was created by protein fold modeling using the MUSTER (MUlti-Source ThreadER) program [28]. The protein model with the lowest level probability density function (PDF) energy was selected. The prepared protein model was energy minimized by the OPLS 2005 force field. Further, the model, thus refined, was validated using ProCheck and Ramachandran plot (RAMPAGE tool; http://raven.bioc.cam.ac.uk/rampage.php).

A molecular docking study was carried out to examine the binding interaction between the modeled C4-LysR protein and 3-HP. The active-site regions in C4-LysR for 3-HP binding were predicted, using the COACH tool, according to the highest C-scores and cluster sizes of 0.05 and 9.0, respectively. The validated model and predicted active-site residues were used to perform docking studies, using the Maestro program from the SCHRODINGER™v10.1 software package to run the docking protocol. Briefly, the target protein (C4-LysR) and the ligand (3-HP) were prepared and processed using Protein Preparation Wizard and LigPrep Wizard in the Schrodinger graphical user interface MAESTRO (version 10.1). Bond orders were assigned to the ligand, and hydrogen bonds consistent with the physiological pH (7.0) were added to the receptor. The initial ligand conformations were obtained by a Monte Carlo conformational search. Using the Receptor Grid Generation tool, a receptor grid box (scaling factor: 1.0; partial charge cutoff: 0.25 Å) was generated around the active-site residues predicted by the COACH tool. Ligand docking was performed using XP (extra precision) predefined docking settings and flexible ligand sampling within the grid box. Finally, the docked poses were visualized using the Maestro 10.1 graphical user interface.

### Analytical methods

The cell concentration was determined in a 10-mm-path-length cuvette using a double-beam spectrophotometer (Lambda 20, Perkin-Elmer, Norwalk, CT). The concentrations of 3-HP were determined by HPLC using a slightly modified version of the method described elsewhere [29]. Briefly, the obtained by 10 min centrifugation of the culture samples at 10,000×*g* was filtered through a Tuffryn-membrane (Acrodisc; Pall Life Sciences, Port Washington, NY) and eluted through a 300 mm × 7.8 mm Aminex HPX-87H (Bio-Rad, USA) column at 65 °C using 2.5 mmol/L H_2_SO_4_ as the mobile phase.

## Additional files

10.1186/s13068-015-0353-5 Comparison of sequence homologies between C3-operon proteins from *P. denitrificans* and other organisms.
10.1186/s13068-015-0353-5 Comparison of sequence homologies between C4-operon proteins from *P. denitrificans* and other organisms.
10.1186/s13068-015-0353-5 Primers used for RT PCR in this study.

## Abbreviations

NAD^+^: oxidized form of nicotinamide adenine dinucleotide
RT-PCR: real-time polymerase chain reaction
*C*_t_: cycle threshold
