# Supplementary material for: Inducible gene expression system by 3-hydroxypropionic acid
Source: Biotechnol Biofuels. 2015 Oct 20;8:169. doi: 10.1186/s13068-015-0353-5 (PMC4617489; doi:10.1186/s13068-015-0353-5)
Supplement: Supplementary file 1 — 10.1186/s13068-015-0353-5 Comparison of sequence homologies between C3-operon proteins from P. denitrificans and other organisms. [file 13068_2015_353_MOESM1_ESM.docx]

**Additional file 1: Table S1.:**

Comparison of sequence homologies between C3-operon proteins from *P. denitrificans* and other organisms.

| **Enzyme Source** | **C3-LysR** | **Identity** | **HpdH** | **Identity** |
| --- | --- | --- | --- | --- |
|  | **Size (AA)** | **(%)** | **Size (AA)** | **(%)** |
| *Achromobacter sp.* | 306 | 44 | 535 | 64 |
| *Acidovorax avenae* | 295 | 59 | 564 | 59 |
| *Acidovorax sp.* | 295 | 44 | 556 | 61 |
| *Acinetobacter baumannii* | 293 | 40 | 534 | 39 |
| *Agrobacterium sp.* | 293 | 42 | 535 | 60 |
| *Alcaligenes faecalis* | 297 | 42 | 555 | 64 |
| *Alcanivorax hongdengensis* | 290 | 27 | 531 | 42 |
| *Alicycliphilus denitrificans* | 304 | 44 | 560 | 60 |
| *Alteromonas marina* | 294 | 36 | 550 | 43 |
| *Azospirillum brasilense* | 291 | 35 | 537 | 36 |
| *Bordetella avium* | 307 | 45 | 540 | 66 |
| *Bradyrhizobium japonicum* | 302 | 41 | 539 | 57 |
| *Burkholderia ambifaria* | 323 | 38 | 537 | 60 |
| *Catenulispora acidiphila* | 299 | 30 | 530 | 37 |
| *Caulobacter sp.* | 305 | 32 | 535 | 59 |
| *Castellaniella defragrans* | 303 | 42 | 537 | 63 |
| *Chromobacterium violaceum* | 305 | 54 | 556 | 68 |
| *Collimonas arenae* | 319 | 44 | 541 | 61 |
| *Comamonas testosteroni* | 300 | 39 | 555 | 59 |
| *Cupriavidus necator* | 296 | 45 | 554 | 61 |
| *Curvibacter gracilis* | 308 | 38 | 575 | 57 |
| *Delftia acidovorans* | 300 | 41 | 575 | 59 |
| *Glaciecola nitratireducens* | 310 | 23 | 533 | 41 |
| *Hahella chejuensis* | 299 | 29 | 558 | 38 |
| *Halomonas elongata* | 315 | 42 | 551 | 61 |
| *Hirschia maritima* | 294 | 29 | 538 | 40 |
| *Idiomarina sp.* | 290 | 27 | 564 | 37 |
| *Janthinobacterium lividum* | 305 | 44 | 541 | 62 |
| *Kitasatospora setae* | 306 | 28 | 529 | 38 |
| *Kutzneria albida* | 324 | 27 | 551 | 41 |
| *Methylobacterium sp.* | 301 | 41 | 546 | 57 |
| *Novosphingobium sp.* | 300 | 40 | 540 | 36 |
| *Paracoccus sp.* | 297 | 36 | 532 | 61 |
| *Parvibaculum lavamentivorans* | 304 | 28 | 548 | 40 |
| *Photobacterium gaetbuleda* | 303 | 26 | 545 | 44 |
| *Polynucleobacter necessarius* | 291 | 41 | 539 | 58 |
| *Pseudogulbenkiania sp.* | 320 | 42 | 550 | 67 |
| *Pseudomonas denitrificans* | 304 | 100 | 554 | 100 |
| *Pseudomonas knackmussii* | 301 | 89 | 552 | 85 |
| *Pseudomonas protegens* | 297 | 71 | 548 | 75 |
| *Pseudomonas fluorescens* | 294 | 72 | 548 | 76 |
| *Pseudoxanthomonas spadix* | 307 | 28 | 545 | 43 |
| *Psychrobacter phenylpyruvicus* | 302 | 25 | 565 | 40 |
| *Rhodomicrobium vannielli* | 296 | 31 | 541 | 43 |
| *Segniliparus rotundus* | 300 | 26 | 516 | 37 |
| *Simiduia agarivorans* | 297 | 28 | 544 | 43 |
| *Sinorhizobium meliloti* | 315 | 37 | 531 | 63 |
| *Sphingobium chlorophenolicum* | 292 | 38 | 544 | 40 |
| *Sphingomonas wittichi* | 294 | 40 | 536 | 57 |
| *Sphingopyxis alaskensis* | 295 | 35 | 528 | 37 |
| *Stenotrophomonas maltophilia* | 289 | 29 | 534 | 44 |
| *Thalassospira xiamenensis* | 295 | 35 | 532 | 62 |
| *Variovorax paradoxus* | 298 | 44 | 544 | 61 |
| *Verminephrobacter eiseniae* | 306 | 28 | 556 | 59 |
| *Vibrio furnissii* | 295 | 27 | 573 | 39 |
| *Xanthobacter autotrophicus* | 307 | 43 | 556 | 56 |
| *Xanthomonas campestri* | 304 | 30 | 556 | 39 |
| *Xanthomonas oryzae* | 304 | 28 | 909 | 38 |
