# Supplementary material for: Inducible gene expression system by 3-hydroxypropionic acid
Source: Biotechnol Biofuels. 2015 Oct 20;8:169. doi: 10.1186/s13068-015-0353-5 (PMC4617489; doi:10.1186/s13068-015-0353-5)
Supplement: Supplementary file 2 — 10.1186/s13068-015-0353-5 Comparison of sequence homologies between C4-operon proteins from P. denitrificans and other organisms. [file 13068_2015_353_MOESM2_ESM.docx]

**Additional file 2: Table S2.:**

Comparison of sequence homologies between C4-operon proteins from *P. denitrificans* and other organisms

| **Enzyme Source** | **C4-LysR** | **Identity** | **MmsA** | **Identity** | **HbdH** | **Identity** |
| --- | --- | --- | --- | --- | --- | --- |
|  | **Size (AA)** | **(%)** | **Size (AA)** | **(%)** | **Size (AA)** | **(%)** |
| *Achromobacter sp.* | 306 | 47 | 497 | 67 | 296 | 56 |
| *Acidovorax avenae* | 295 | 59 | 507 | 82 | 299 | 54 |
| *Acidovorax sp.* | 301 | 60 | 507 | 82 | 296 | 55 |
| *Acinetobacter baumannii* | 293 | 49 | 505 | 70 | 296 | 59 |
| *Aeromonas hydrophilia* | 304 | 36 | 503 | 58 | 306 | 55 |
| *Agrobacterium sp.* | 293 | 37 | 518 | 47 | 294 | 45 |
| *Alcaligenes faecalis* | 297 | 45 | 497 | 60 | 298 | 55 |
| *Alcanivorax hongdengensis* | 302 | 39 | 498 | 56 | 287 | 48 |
| *Alicycliphilus denitrificans* | 304 | 58 | 505 | 81 | 298 | 53 |
| *Alteromonas marina* | 294 | 35 | 496 | 48 | 291 | 62 |
| *Anaeromyxobacter* | 313 | 31 | 491 | 53 | 293 | 29 |
| *Azospirillum brasilense* | 291 | 33 | 499 | 51 | 296 | 53 |
| *Azotobacter vinelandii* | 296 | 72 | 501 | 92 | 297 | 79 |
| *Beijerinckia indica* | 301 | 43 | 509 | 50 | 295 | 52 |
| *Bordetella avium* | 307 | 48 | 497 | 66 | 294 | 58 |
| *Bradyrhizobium japonicum* | 302 | 42 | 498 | 49 | 296 | 50 |
| *Burkholderia ambifaria* | 319 | 47 | 509 | 74 | 300 | 65 |
| *Catenulispora acidiphila* | 296 | 35 | 504 | 42 | 301 | 41 |
| *Caulobacter sp.* | 295 | 31 | 498 | 45 | 295 | 43 |
| *Castellaniella defragrans* | 303 | 46 | 497 | 64 | 297 | 59 |
| *Chromobacterium* | 305 | 41 | 500 | 79 | 296 | 58 |
| *Collimonas arenae* | 319 | 47 | 502 | 67 | 297 | 54 |
| *Comamonas testosteroni* | 300 | 54 | 507 | 83 | 298 | 52 |
| *Corynebacterium* | 304 | 28 | 504 | 51 | 291 | 42 |
| *Cupriavidus necator* | 308 | 40 | 507 | 73 | 296 | 66 |
| *Curvibacter gracilis* | 296 | 60 | 505 | 82 | 294 | 54 |
| *Delftia acidovorans* | 300 | 54 | 507 | 82 | 298 | 53 |
| *Ferrimonas balearica* | 284 | 25 | 497 | 55 | 296 | 51 |
| *Glaciecola nitratireducens* | 281 | 28 | 496 | 56 | 295 | 47 |
| *Gordonia bronchialis* | 298 | 32 | 513 | 48 | 289 | 46 |
| *Hahella chejuensis* | 302 | 28 | 498 | 51 | 296 | 51 |
| *Halomonas elongata* | 315 | 44 | 499 | 67 | 300 | 53 |
| *Hirschia sp.* | 294 | 37 | 498 | 43 | 293 | 45 |
| *Idiomarina sp.* | 312 | 28 | 499 | 57 | 297 | 52 |
| *Janthinobacterium lividum* | 305 | 46 | 502 | 75 | 297 | 53 |
| *Kitasatospora setae* | 304 | 31 | 508 | 43 | 298 | 40 |
| *Kutzneria albida* | 300 | 35 | 501 | 45 | 284 | 44 |
| *Methylobacterium sp.* | 302 | 41 | 499 | 47 | 297 | 47 |
| *Methylocystis sp.* | 294 | 30 | 498 | 48 | 295 | 46 |
| *Novosphingobium sp.* | 316 | 39 | 499 | 45 | 289 | 45 |
| *Oceanimonas smirnovii* | 288 | 28 | 497 | 58 | 297 | 47 |
| *Paracoccus sp.* | 297 | 38 | 533 | 46 | 302 | 45 |
| *Parvibaculum* | 304 | 30 | 500 | 52 | 296 | 57 |
| *Phenylobacterium* | 282 | 32 | 498 | 52 | 298 | 49 |
| *Photobacterium* | 303 | 26 | 502 | 53 | 303 | 44 |
| *Polynucleobacter* | 291 | 49 | 500 | 79 | 298 | 66 |
| *Pseudoalteromonas* | 299 | 29 | 496 | 55 | 299 | 52 |
| *Pseudogulbenkiania sp.* | 320 | 46 | 500 | 79 | 298 | 59 |
| *Pseudomonas denitrificans* | 298 | 100 | 501 | 100 | 291 | 100 |
| *Pseudomonas knackmussii* | 298 | 95 | 504 | 93 | 291 | 92 |
| *Pseudomonas protegens* | 316 | 45 | 508 | 73 | 295 | 62 |
| *Pseudomonas fluorescens* | 315 | 45 | 505 | 73 | 295 | 60 |
| *Pseudoxanthomonas spadix* | 297 | 27 | 501 | 79 | 297 | 57 |
| *Psychrobacter* | 302 | 27 | 495 | 71 | 314 | 52 |
| *Ralstonia oxalatica* | 298 | 30 | 515 | 73 | 301 | 65 |
| *Rhodomicrobium vannielli* | 296 | 30 | 496 | 48 | 296 | 48 |
| *Segniliparus rotundus* | 300 | 25 | 509 | 51 | 300 | 46 |
| *Shewanella oneidensis* | 291 | 24 | 499 | 55 | 300 | 51 |
| *Simiduia agarivorans* | 297 | 29 | 505 | 55 | 296 | 47 |
| *Sinorhizobium meliloti* | 315 | 40 | 498 | 50 | 298 | 52 |
| *Sphingobium* | 292 | 43 | 499 | 49 | 294 | 48 |
| *Sphingomonas wittichi* | 325 | 43 | 503 | 44 | 296 | 46 |
| *Sphingopyxis alaskensis* | 310 | 36 | 497 | 45 | 291 | 44 |
| *Stenotrophomonas* | 289 | 32 | 501 | 80 | 296 | 57 |
| *Tatlockia micdadei* | 293 | 22 | 499 | 45 | 295 | 47 |
| *Thalassospira xiamenensis* | 295 | 38 | 499 | 45 | 296 | 48 |
| *Variovorax paradoxus* | 298 | 60 | 507 | 82 | 300 | 55 |
| *Verminephrobacter* | 298 | 26 | 507 | 78 | 299 | 51 |
| *Vibrio furnissii* | 304 | 25 | 520 | 57 | 300 | 49 |
| *Xanthobacter* | 307 | 44 | 498 | 50 | 299 | 51 |
| *Xanthomonas campestri* | 301 | 29 | 501 | 77 | 295 | 58 |
| *Xanthomonas oryzae* | 304 | 27 | 501 | 77 | 300 | 57 |
