# Supplementary material for: Inducible gene expression system by 3-hydroxypropionic acid
Source: Biotechnol Biofuels. 2015 Oct 20;8:169. doi: 10.1186/s13068-015-0353-5 (PMC4617489; doi:10.1186/s13068-015-0353-5)
Supplement: Supplementary file 3 — 10.1186/s13068-015-0353-5 Primers used for RT PCR in this study. [file 13068_2015_353_MOESM3_ESM.docx]

**Additional file 3: Table S3.:** Primers used for RT PCR in this study

| Primer (Forward, FP; Reverse, RP) | Sequence 5’ to 3’ |
| --- | --- |
| *Pseudomonas denitrificans* |  |
| Pd rpoD, FP | TCCGGTGCACATGATCGA |
| Pd rpoD, RP | GATCTTGTCTTCCGGCATGTC |
| Pd hpdH, FP | GGACAACTATCCCTGGATCCACAT |
| Pd hpdH, RP | GTATTTGATCGACCGGCCATT |
| Pd mmsA, FP | ACGTGGTGAACGCGATCTG |
| Pd mmsA, RP | GTTCTTCGCCCCCATCATG |
| Pd hbdH-4, FP | CGATGACGTCGAGGTGATCA |
| Pd hbdH-4, RP | GATGGTGGACGAGTCGATCAG |
| *Pseudomonas knackmussii* |  |
| Pk rpoD, FP | CTTACGCGGAGGTCAACGA |
| Pk rpoD, RP | GAATACGTTGATCCCCATGTCAT |
| Pk hpdH, FP | CGACAACGAAGGCTGTGGATA |
| Pk hpdH, RP | GGTTCGGGCGATCCATGA |
| Pk mmsA, FP | ACGTGGTGAACGCGATCTG |
| Pk mmsA, RP | TTCTTCGCGCCCATCATG |
| Pk hbdH-4, FP | GCCAAGGTGGCCAACAACTT |
| Pk hbdH-4, RP | GCCAGTACTTCCGCATCCAT |
| *Pseudomonas fluorescens* |  |
| Pf rpoD, FP | AGCTGGTGCCCAAGCAATT |
| Pf rpoD, RP | CGCACACAGAGCTGCATGA |
| Pf hpdH, FP | GGCCAAGGCGTTTCTCAAG |
| Pf hpdH, RP | GCAAGCACGCCCGTTCT |
| Pf mmsA, FP | AGCCTTGATCCGCGAACAT |
| Pf mmsA, RP | CCAGGCCACGGAAAATATCG |
| Pf hbdH-4, FP | GTGTGGCTGAATGAAGACGGTGTA |
| Pf hbdH-4, RP | CCGCTACTTCACGGATGGTTT |
| *Alicycliphilus denitrificans* |  |
| Ad rpoD, FP | ACCCGGCTGCTGGAAAAG |
| Ad rpoD, RP | CGTAGCCCTCCTTGTCGTAGA |
| Ad hpdH, FP | GGCCAAGATCTGCAACAACA |
| Ad hpdH, RP | CATGATCTCGCTGAGCACCTT |
| Ad mmsA, FP | CGACCACCCGGACATCAA |
| Ad mmsA, RP | GTTCTTCGCGCCCATCAT |
| Ad hbdH-4, FP | GCTACTTCGAGGTCAACCAGAAG |
| Ad hbdH-4, RP | CTGGGCAGAGGTCCACAACT |
| *Pseudogulbenkiania sp.* |  |
| P rpoD, FP | ACGCCAATCTGGAAGAACTCAA |
| P rpoD, RP | TCGCTCTGGTTACCCTGCTT |
| P hpdH, FP | CGCAATTGCCTGCCTTACTTC |
| P hpdH, RP | GTACAGCGGGTTCTGCATGTT |
| P mmsA, FP | GCCAAGACGGCTTTCAAGAG |
| P mmsA, RP | CGCCAGTTCCTTCATGTGTTC |
| P hbdH-4, FP | CCAAGATCTGCAACAACATGCT |
| P hbdH-4, RP | CCCGAACTCTTGGCGATGA |
| *Acidovorax sp.* |  |
| A rpoD, FP | GGCCTGCAGTTCCTGGATCT |
| A rpoD, RP | TGGCGTAGGTCGAGAACTTGT |
| A hpdH, FP | GTGCCCACACTGAACATGCT |
| A hpdH, RP | CGACGGTGCCATGCTCAT |
| A mmsA, FP | ATCCACGGCGGTGAAGAC |
| A mmsA, RP | GGCGCGGTTGTACACATG |
| A hbdH-4, FP | GCCAGCCAACACGTGGAA |
| A hbdH-4, RP | GGCGGCGGCTATTGTTGA |
| *Achromobacter xylosoxidans* |  |
| Ax rpoD, FP | CGCAGGAGTCGATCCAGAAC |
| Ax rpoD, RP | GTTCGAGCTGGCGCACTT |
| Ax hpdH, FP | CGCGATCACGCCGAACTA |
| Ax hpdH, RP | TTCCTCGGGCTGGTAACG |
| Ax mmsA, FP | CCGCGTTCCGCTATTGAT |
| Ax mmsA, RP | GCACCTTGGCCACGACTTC |
| Ax hbdH-4, FP | GCCGAGGTGGTGATCTCGAT |
| Ax hbdH-4, RP | CGATGGTGCTGCACTCGAT |
| *Acidovorax avenae subsp. Citrulli* |  |
| Ac rpoD, FP | AGGAACGCGCCTTGATGA |
| Ac rpoD, RP | TCAGGTAGCCACGGGTCTTG |
| Ac hpdH, FP | GACACCTCTTTCGACTTCATCGT |
| Ac hpdH, RP | GGGAATGTGGATCCAGTGGTA |
| Ac mmsA, FP | GCATCACGCCGTTCAACTT |
| Ac mmsA, RP | GTCCTGCTCGCTGGGTTTG |
| Ac hbdH-4, FP | GAACTGGGCCCTGGAAAAATAC |
| Ac hbdH-4, RP | TTCACCGCAGCAGCGTTCT |
| *Variovorax paradoxus* |  |
| Vp rpoD, FP | AGGAACGCGCCTTGATGA |
| Vp rpoD, RP | CGAGCGTGATCAGCGTCTTC |
| Vp hpdH, FP | GCATCAACGCCACCATCTG |
| Vp hpdH, RP | ATGCCGAGGCAGTGCTCATA |
| Vp mmsA, FP | GGCATCACGCCGTTCAACT |
| Vp mmsA, RP | TCTTGCTCGGAAGGCTTCAG |
| Vp hbdH-4, FP | GGGCGCCAACATCTTCCAT |
| Vp hbdH-4, RP | CTCCGAGGTGCCGATCATC |
